# Earth observation data uncover green spaces’ role in mental health

**DOI:** 10.1038/s41598-024-72008-8

**Published:** 2024-09-09

**Authors:** Leonardo D. Araújo, Daniel C. Zanotta, Nicolas Ray, Maurício R. Veronez

**Affiliations:** 1 Laboratory of Advanced Visualization and Geoinformatics (VizLab), Universidade Do Vale Do Rio Dos Sinos, Av. Unisinos 950, Cristo Rei, São Paulo, RS 93022-750 Brazil; 2 Geo Health GroupInstitute of Global Health, Faculty of Medicine, University of Geneva, Chemin des Mines 9, 1202 Geneva, Switzerland; 3 Institute for Environmental Sciences, University of Geneva, 66 Boulevard Carl-Vogt, 1205 Geneva, Switzerland

**Keywords:** Ecology, Environmental sciences, Epidemiology

## Abstract

The prevalence of mental health disorders, a key disability cause, is linked to demographic and socioeconomic factors. However, limited data exists on mental health and the urban environment. Urbanization exposes populations to environmental stressors, particularly affecting low-middle-income countries with complex urban arrangements. We used remote sensing and census data to investigate potential connections between environmental factors and mental health disorders. Land cover variables were assessed using the European Space Agency (ESA) global WorldCover product at 10 m resolution together with the database of mental health diagnosed cases (n = 5769) from the Brazilian Unified Health System’s Department of Informatics (DATASUS) from every health facility of the city of Porto Alegre. The association of mental health data with land cover was established with machine learning algorithms and polynomial regression models. The results suggest that higher trees cover at neighborhood level was associated with better mental health index. A lower mental health index was also found to be associated with an higher Human Development Index. Our results highlight the potential of greenness in the city environment to achieve substantially better mental health outcomes.

## Introduction

Earth observation (EO) through remotely sensed data has emerged as a valuable tool in consistently measuring spatial and temporal attributes relevant to the achievement of Sustainable Development Goals^1^. In addition to its applications in environmental monitoring^2^ and resource management^3^. EO-derived data also holds promise in addressing gaps in public health and well-being indicators^4^. This is especially pertinent in the context of mental health disorders, which pose significant public health challenges on a global scale^5^. Therefore, leveraging the power of EO data offers a pathway to the environmental determinants of mental health and contributes to more effective public health interventions.

The prevalence of mental health disorders appears to be increasing in contemporary societies. Depression, which impacts around 264 million people globally and stands as a leading cause of disability, along with anxiety disorders that affect a similar number of people. There is a notable degree of overlap between the two conditions^6^, contributing significantly to the Global Burden of Disease (GBD)^7^. According to data from the 2019 Global Health Data Exchange, approximately 3.8% of the world’s population had mental health disorders at that time^8^, and in Brazil, the prevalence was estimated at 4.3%^9^. The association of mental health cases with a range of factors, including social, economic, physiological, behavioural, genetic and cultural causes is well-established^10^, but limited quantitative data exist on the relationship between mental health and the urban environment. As urban growth expands, especially in cities in low and middle-income countries (LMICs), urban areas are grappling with rate of population growth that surpasses the pace of infrastructure expansion^11^. This can lead to more informal settlements^12^, usually situated in geographically and environmentally dangerous areas, characterized by escalating exposure to environmental stressors, potentially amplifying stress levels and compromising mental well-being^13^. Mental health and environmental degradation are global challenges, and an increasing body of research literature has explored the connections between them^14^. Environmental degradation and the loss of livelihood due to changing climatic conditions can increase depression, post-traumatic stress disorder (PTSD), and enduring grief^15^. Consequently, it becomes indispensable to identify specific urban environmental features that influence the health of city dwellers.

The natural environmental context in which people live plays an important role in mental health^16^ and in some cases can mitigate some of the above-mentioned adverse health impacts found in of LMICs cities^17^. Several studies have reported an association between neighbourhood greenness and with good mental health in adults^18^, reduced prevalence of depression^19^ and improved measures of mental well-being such as quality of life^20^ and life satisfaction^21^. Besides, moving to greener areas improves mental health^22^ and these areas have lower rates of prescriptions for psychotropic medications for anxiety, depression, and psychosis than urban areas^23^.

Greenness levels are intrinsically linked to various critical ecosystem services, including medicinal resources, food supply, and potable water. Additionally, these green areas serve as natural buffers, mitigating the detrimental impacts of environmental stressors and can act as regulation services such as control of air and noise pollution, as well as moderation of extreme heat^24^.

Around the world, mechanisms for studying the relationship between green areas and mental health are diverse, as are the mechanisms by which green areas promote good mental health. One early theory presented by Kaplan and Kaplan suggests that having access to green areas in urban environments facilitates “psychological restoration” countering the mental fatigue caused by modern living^25^. It has been shown that being close to green spaces can improve psychological health in various ways, such as reducing cortisol levels^26^, buffering the adverse effects of stressful life events^27^, fostering social cohesion^28^ and enhancing overall psychological well-being^29,30^. These findings underscore the significance of urban green spaces in promoting mental health and well-being, contributing positively to the quality of life in cities. Urban green spaces foster improved mental health by people simply by being present, nearby, or in view^28^, facilitating the restoration of depleted cognitive capacities^31^, aiding in the recovery from periods of psychosocial stress^32^, and promoting increased optimism^33^. The enhancement of these mental health advantages might be due in part to natural, biodiverse sound environments that calmly reduce chronic noise, and potentially mitigate the impact of socioeconomic disadvantage on mental well-being^34^, but also to social and physical activities enabled by nearby green spaces^35^.

For mental health, assessment techniques can include self-reported measures^36^, as well as subjective well-being indexes^37^. Studies evaluating of green spaces in relation to mental health have used satellite imagery to derive Normalized Difference Vegetation Index (NDVI)^38^, the use of geo-tagged street view images^39^, and other types of Earth observation data^40,41^. Furthermore, diverse indices have been used in prior research to evaluate exposure to urban green spaces, including Land Use/Land Cover (LULC) classifications derived from satellite imagery^41,42^, which has been shown to play a significant role in mental health outcomes. However, there are few studies addressing the relationship between different classes of LULC and mental health in LMICs, as the presence of green spaces has a very irregular distribution, availability, quality and use in these regions^43^. Recently, a potential upward trend in the benefits of urban green spaces was found among people from lower socioeconomic classes in LMICs^38,44^. For example, in the Sao Paulo Metropolitan Area, the 6th most populated city in the world with around 21 million inhabitants, the prevalence of anxiety was negatively correlated with the presence of green spaces, predominantly grassy areas, and positively correlated with roofs and asphalt^41^.

Currently, there exists a lack of comprehensive information regarding the influence of different land use categories on the mental well-being of individuals in the southern region. The authorities have focused on emergency initiatives and symptomatic solutions that are temporary, costly, and often not effective. Given these prevailing circumstances, it is imperative to better comprehend the relationship between various land use patterns, specifically focusing on the presence of green spaces within urban settings and their potential impact on the mental health of the local population. These recommendations should delineate areas where mental health requires heightened attention, as well as pinpoint potential locations for strategic LULC interventions. Such insights are intended to provide valuable guidance to policymakers in shaping future urban management strategies for the city, as well as recommendations to health authorities. In this context, the aim of this study is to examine the connections between Land Use/Land Cover (LULC) and mental health outcomes within the population of Porto Alegre, a city located in southern Brazil with a predominantly low to middle-income demography.

## Results

### Spatial autocorrelation of mental health cases

A total of 5,769 diagnosed cases of mental health issues were included in the study with initial diagnostic from January 2018 to July 2023. The Global Moran’s I spatial statistical analysis (Moran’s Index = 0.072, *p*-value = 0,000 and Z-score = 3706) indicates there is less than 1% likelihood that this clustered pattern results from random chance. The positive value of the Moran’s Index indicates clustering effects in the mental health case distribution in the city of Porto Alegre, i.e., the presence of similar clusters close to each other in the data suggests the presence of spatial distribution patterns.

### Hot spot analysis

**Fig. 1:**
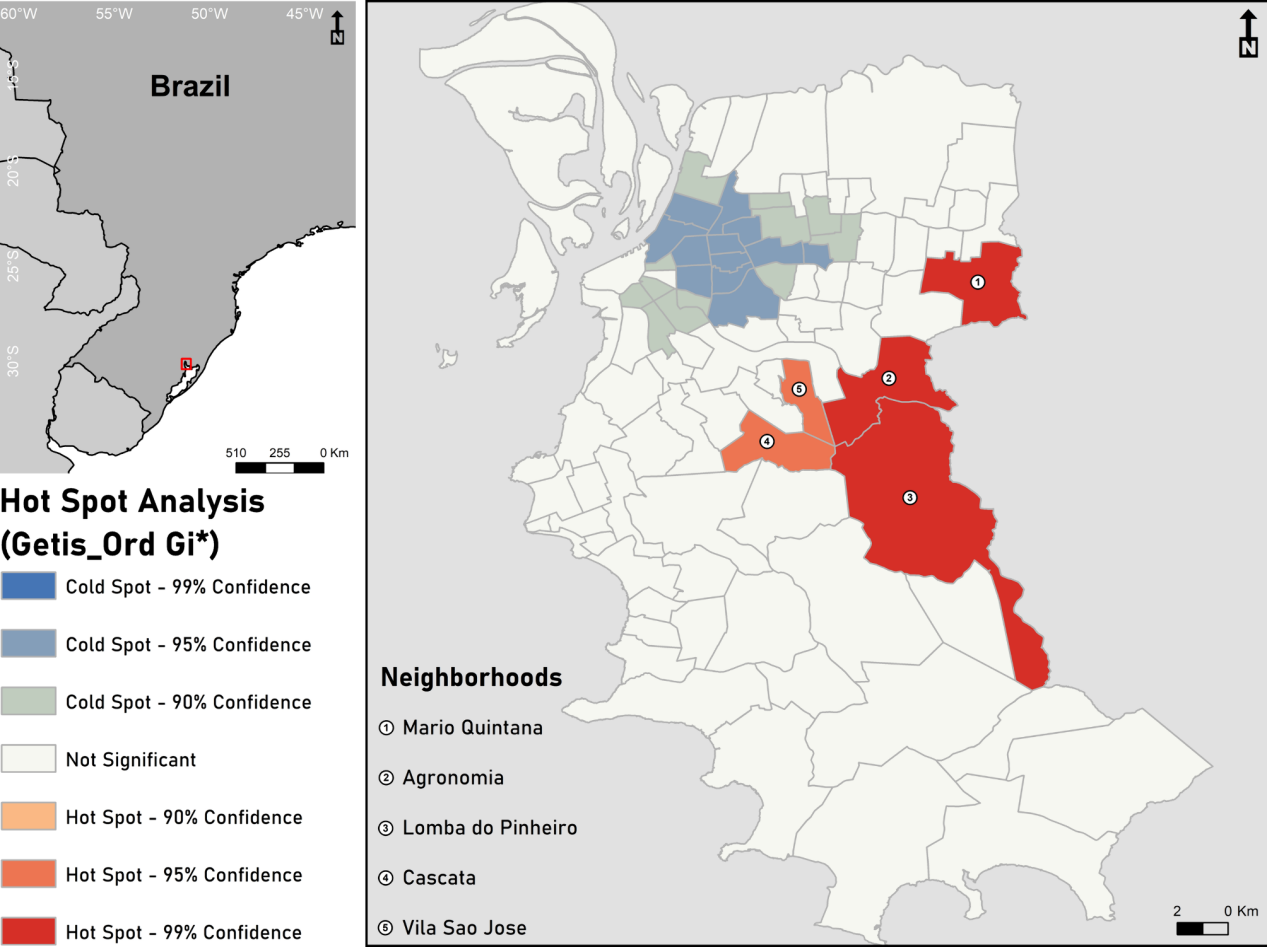
Hot spot analysis of mental health cases in Porto Alegre, where red colours represent high rate of mental health cases and blue colors low rates (cold spot area), generated in ArcMap® 10.6.1^69^.

Figure 1 presents the map of the hotspot analysis for the study area that highlights the neighborhoods with high (hot spot) or low (cold spot) proportion of mental health cases. Risk areas are represented by red colours (high rate of mental health cases). The hot spot areas are in Mario Quintana (northeast) and Agronomia, Lomba do Pinheiro, Cascata and Vila São José, mainly in the east zone. Five hotspots are identified and represent 19.93% of the total diagnostic cases. The total area of hotspots is 54,600  km^2^, which equals 11.02% of the city area. The five hotspot neighborhoods are Mario Quintana (Z-score = 2655; *p*-value = 0.007), Agronomia (Z-score = 3,089; *p*-value = 0,002), Lomba do Pinheiro (Z-score = 4,190; *p*-value = 0,000), Cascata (Z- score = 2508; *p*-value = 0.012) and Vila Sao Jose (Z-score = 2,036; *p*-value = 0,041). The larger the Z-score and the lower the p-value, the more intense the clustering at that location.

### Land use/land cover and mental health index

**Fig. 2:**
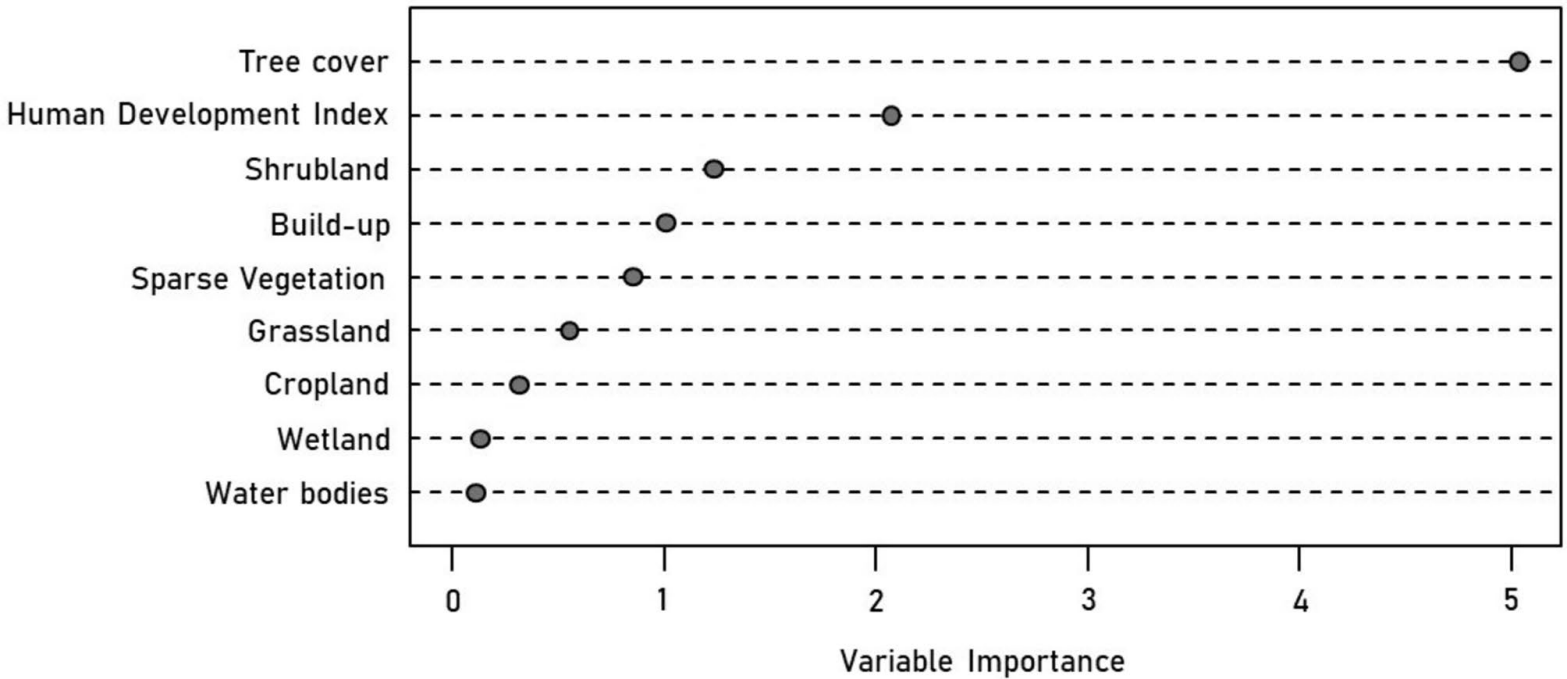
Ranking of LULC variables according to their importance, as estimated by Random Forests, to predict the mental health index in Porto Alegre neighbourhoods.

**Fig. 3:**
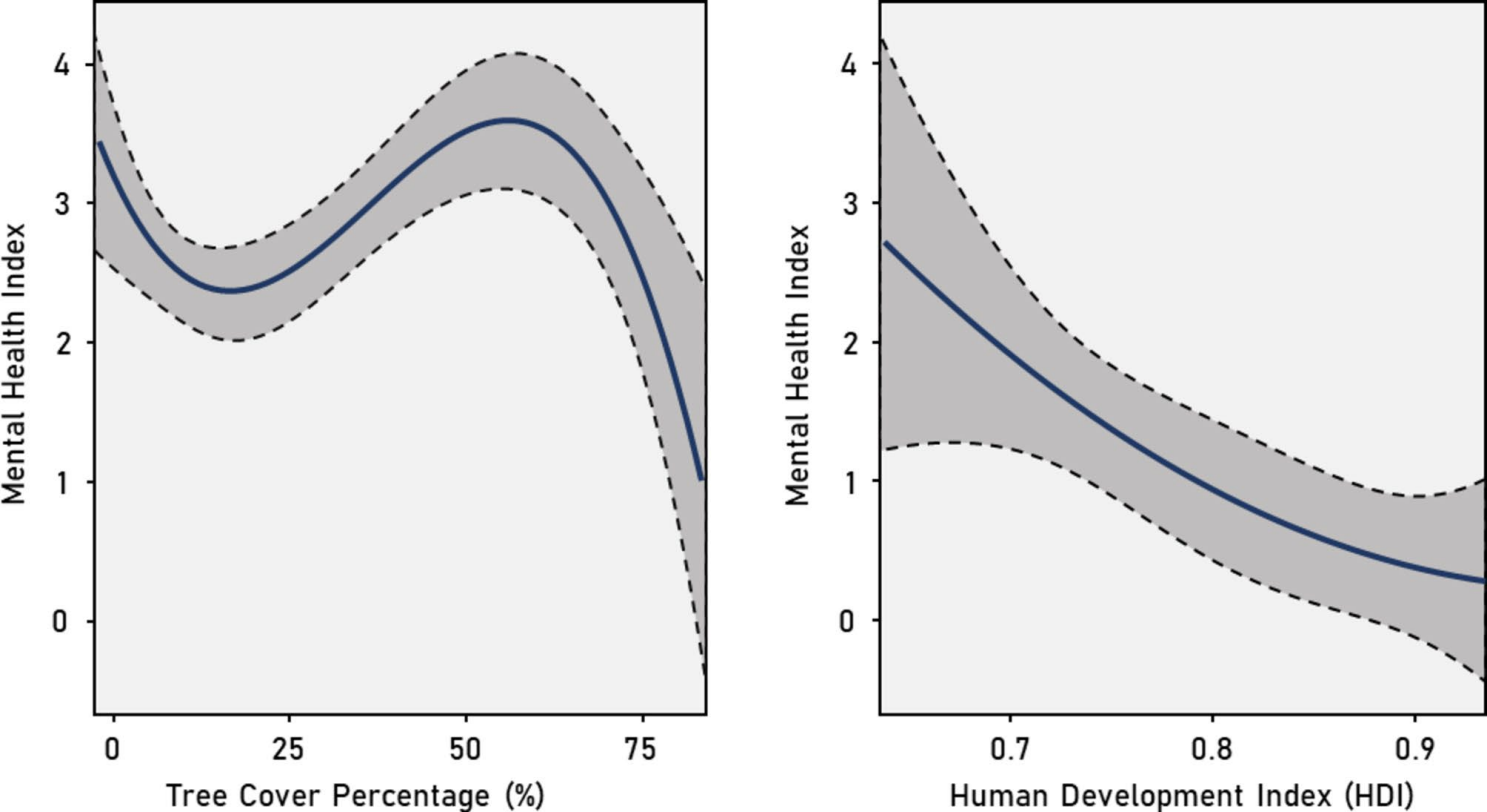
Fitted curves for predicting the Mental Health Index (MHI) in response to significant predictors, which is: Tree cover and Human Development Index. The curve (continuous black line) and the 95 confidence bands (shaded areas) were obtained by fitting the Mental Health Index as function of LULC variables using Polynomial Regression.

Figure 2 summarizes the importance of each LULC variable for predicting the Mental Health Index. The goodness-of-fit and accuracy metrics of the Random Forest algorithm are MSE: 0.03, RMSE: 0.19 and MAE: 0.14. The Tree cover percentage and the Human Development Index are the most important predictors (IncNodePurity = 5.03 and 2.07, respectively) of the Mental Health Index in the neighborhoods. The metrics of the Polynomial Regression model are AIC: − 10.43, BIC: 7.29 and Residual std. error: 0.21. The fitted curves in Fig. 3 confirm the direct and indirect effects of the Tree cover percentage and the Human Development Index on the Mental Health Index. Higher Tree cover and the Human Development Index are associated with a reduction of the Mental Health Index in the neighborhoods.

## Discussion

Our study investigated the spatial distribution of diagnosed mental health cases in Porto Alegre and their associations with various remotely sensed Land Use/Land Cover (LULC) variables and the Human Development Index (HDI) using a machine learning and polynomial regression model approach. The spatial distribution of diagnosed mental health cases exhibited significant variations across neighborhoods, as well as significant clustering. The latter may reflect socio-economic conditions, other shared characteristics of the communities living in these areas, or similar environmental conditions and stressors. Our machine Learning analysis of the different LULC classes and HDI revealed statistically significant associations with mental health cases. In this study, the LULC class Tree cover and the HDI variable emerged as the most relevant predictors of our Mental Health Index. The Polynomial Regression model demonstrated a negative correlation between the most significant predictors and the Mental Health Index. Specifically, an increase in neighborhood Tree cover above 50% was associated with a decrease in the Mental Health Index. A similar trend was observed for the HDI, where higher neighborhood HDI values (from 0.7 upward) corresponded to lower values of the Mental Health Index. To the best of our knowledge, our study is the first one to test directly, through machine learning and a modelling technique, the influence of different LULC classes and the HDI on the number of diagnosed on mental health cases in a low- to middle-income city.

In Fig. 3 the beginning of the tree cover percentage plot presents a momentary decrease. The areas with low tree cover are typically correspond neighborhoods that are very densely populated and tend to present squares or small groves that were planned to be used mainly for relaxation and rest, indicating that these structures are correlated with lower MHI. The middle of the curve (from about 30% to 55% tree cover) is more related to neighborhoods featuring wastelands and abandoned places, which are areas where the level of greenness is not conducive to generating mental health benefits. At last, the curve presents a high decrease, which is correlated with large parks and even urban forests, areas know to provide mental health benefits. The Tree cover class could be used as a good proxy for green spaces. This class refers to the clustering of tall (15 m or higher) dense vegetation, typically with a closed or dense canopy and has high statistical accuracy^45^. Recent studies have reported that the presence of green spaces is linked to various advantageous health outcomes, including negative associations with the presence of anxiety^41^. Changes to ecosystems resulting from alterations in land use can profoundly impact mental well-being, as they are intricately linked to both individual and community identity and often form an essential component of culture and overall sense of well-being^46,47^. Our results are in line with these earlier works.

Previous research has explored the effects of ethnicity and socioeconomic factors influencing mental health outcomes^48^. However, quality and accessibility of those types of data are usually low, especially in lower-income communities^49^. The Brazilian Public Unified Health System (SUS) runs the Department of Information Technology (DATASUS) that is responsible for developing, researching, and integrating information technologies to support healthcare systems^50^. DATASUS maintains the systems and applications necessary to record, process, and make available health information^51^, but despite the national consensus about its importance as an instrument of health surveillance, there is around 17.7% of unreported information^52^, making it a challenge for studies such ours. The use of HDI in our model mitigated the lack of reliable national social-economic data in Brazil, and HDI was shown to be one of the most important predictors together with the Tree cover. Our finding that as the HDI increases, the MHI tends to decrease, is in line with previous studies, notably a multi-country study that found that countries with a low HDI had the highest prevalence of depression^53^. A similar study done in Asia^54^ showed that depressive disorder had a significant economic burden on individuals and society, which could be a consequence of deprivation^54–57^.

Our findings also contribute to the growing literature on green space and the Human Development Index on mental health disorders. Our results are consistent with the generic research findings of the health benefits of living in areas with high levels of green space, but several limitations of our study should be acknowledged. Firstly, our research was limited by the lack of information on the severity levels of depression and anxiety. As a result, we were unable to determine how these conditions, in relation to Land Use/Land Cover, are impacted at specific severity levels. Secondly, we determined vegetation density utilizing aerial measures rather than eye-level or other ground-level assessments. This may offer different perceptions of green spaces compared to eye-level evaluations^58^. Thirdly, our study did not consider the physical accessibility or usability of green spaces, such as parks and squares, factors that could significantly contribute to improving mental and overall health. Recent investigations have crafted methodologies to model physical access to urban green spaces, using strategies that account for movement barriers and walking speeds^59,60^. In future studies, these approaches could be used with ours to explore if distance to green spaces, as well as frequency of use and type of interaction with these spaces, is associated with certain mental health conditions. Lastly, our assessment of mental health cases was based on clinician-rating scales through self-reported instruments. While certain symptoms might be better suited for self-reporting, this approach can be influenced by potential biases when patients describe their symptomatology, personality traits, and demographic characteristics such as education, ethnicity, and socioeconomic factors. Despite these potential biases, self-reporting has, in some instances, proven to be an effective mechanism for assessing mental health disorders, such as depression^61^.

Moreover, when assessing the impact of green spaces on human well-being, it is crucial to recognized the disproportionate burden faced by marginalized communities, particularly those with lower socioeconomic status (SES), including low-income individuals and racial or ethnic minorities. These vulnerable populations are often subjected to living in polluted and deteriorated environments, characterized by limited access to quality green spaces, higher exposure to environmental hazards, and inadequate infrastructure^62^. These population may also be more prone to the impacts of future climatic extremes^63^, and the relationship between increased or decreased local urban temperature and mental health should be explored in futures studies.

Finally, our data suggest that the presence of mental health disorders in the city of Porto Alegre was associated with decreased density of tree cover. Residing in areas with a high level of greenness and a high Development Index is associated with lower instances of mental health issues in the urban environment of Porto Alegre. For Porto Alegre, it is advised to carry out and effective tree monitoring maintenance plan based on the Plan Director of Arborization of the municipality, and to create effective guidelines regarding the city’s tree. Priority should be given to the hotspot neighborhoods identified in our study. Despite the challenges of significantly increasing green spaces in densely populated city areas, these results remain relevant for policymaking, advocating the potential health benefits that could be achieved through improved urban planning and development initiatives.

## Methods

This study was conducted with the approval of the Ethics Research Committee of Unisinos University, under number 61224822.6.0000.5344.

### Study area

**Fig. 4:**
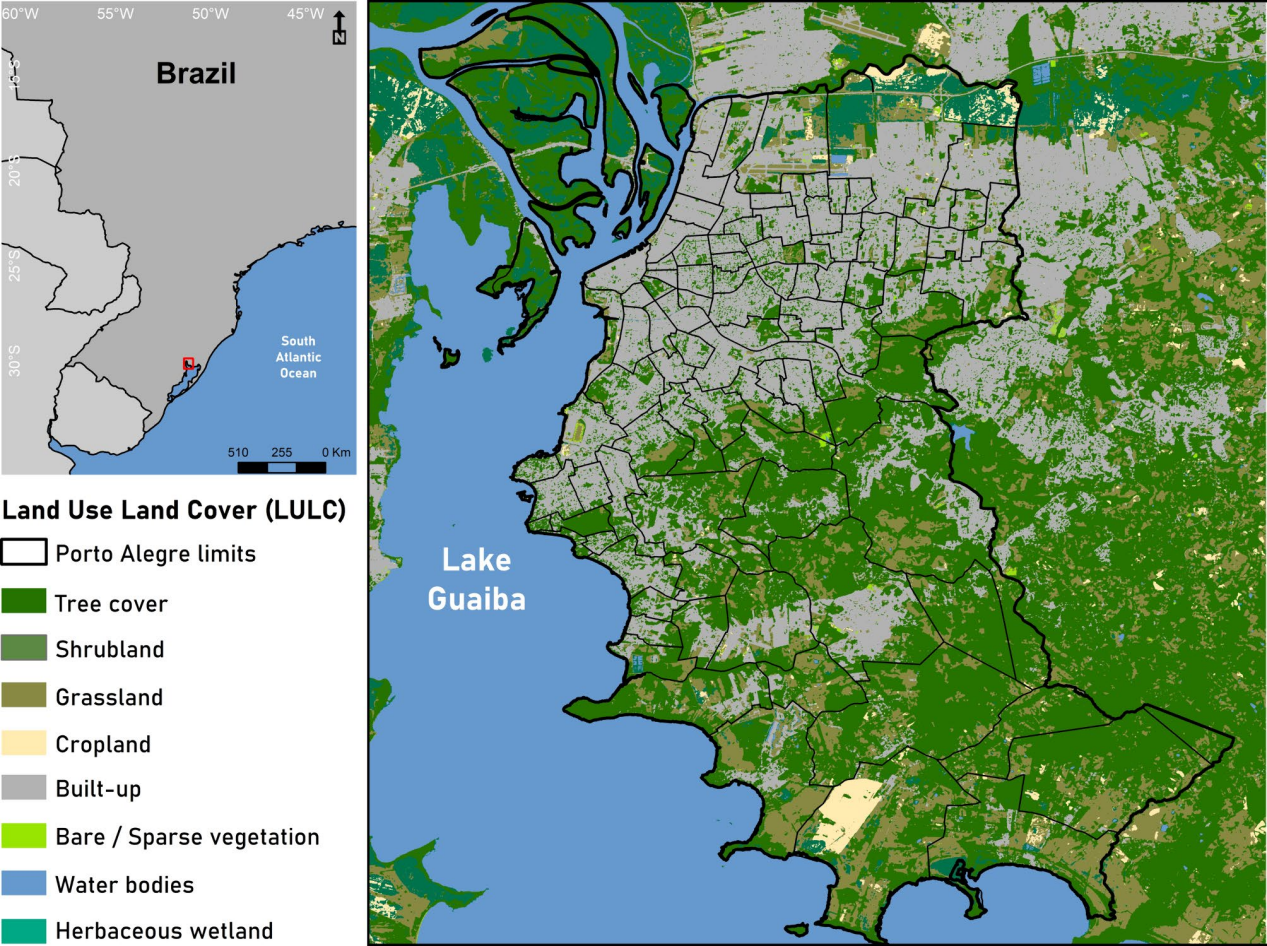
The study area map shows Porto Alegre in the left corner in relation to Brazil and in the right, the Land Use/Land Cover (LULC) of the city borders, generated in ArcMap® 10.6.1^69^.

The study was conducted in the low-middle-income city of Porto Alegre, southern Brazil, situated at 30°1′40″ south and 51°13′43″ west (Fig. 4) covering an area of approximately 495,390 km^2^, divided into 94 neighbourhoods, with a population of approximately 1,332,570 inhabitants in the 2023 demographic census^64^. Porto Alegre has a humid subtropical climate, with four well-defined seasons and significant climate variability^64^. The city has an annual average temperature of 19.5 °C and an average annual rainfall of 112 mm. According to the Brazilian Institute of Geography and Statistics, Porto Alegre has a Human Development Index (HDI) of around 0.805^65^, classified as very high according to the criteria of the United Nations Development Program^66^. According to the World Health Organization, for a city to be considered well-forested, the recommended minimum green area per inhabitant is approximately 12 m2. With about 49 m2 of green areas per inhabitant, Porto Alegre is considered one of the most forested capitals in Brazil, ranking fourth in urban afforestation, with 82.9% of the area covered^65^. On the other hand, Porto Alegre leads the diagnoses of depression and suicide cases among the capitals of Brazil, the Brazilian average is about 11.3% cases per 100,000 inhabitants, while Porto Alegre has a rate of about 17.5%^67^.

### Mental health index

Mental health diagnosticated cases were accessed through the Brazilian Public Unified Health System (SUS) from the city of Porto Alegre. The database includes cases of the Notifiable Diseases Information System (SINAN) from the Ministry of Health, Authorization for Hospitalization in Public Hospitals (AIH), and Primary Health Care (APS, with 132 Health Units). The International Statistical Classification of Diseases and Related Health Problems^68^ was used, specifically the ICD codes F31 and F42. The data was collected precisely for the resident population of Porto Alegre and the address of each mental health diagnosticated case was georeferenced in the ArcGIS software^69^. The data spanned from January of 2018 to July of 2023 and due to the lack of georeferencing limitations (missing address on the database) and duplicate records, our analysis evaluated a sample of 5,769 cases. Subsequently, we create a Mental Health Index (MHI) adapted from the descriptive measures of incidence and prevalence which have been largely used in epidemiology^70^. Here, the Mental Health Index was based on the number of cases divided by the population of each one of the 94 neighbourhoods. To calculate the MHI for each of the neighborhoods, we divided the number of cases at neighborhood level by the number of populations of each neighbourhood and then multiplied by 100. This ensured our continuous dependent variable to be test against the Land Use/Land Cover variables and the Human Development Index.

### Human development index

In this study, we considered the Human Development Index (HDI)^66^ as a proxy for demographic and socioeconomic conditions, due to known bias in Brazilian studies when collecting income and social data. The HDI is calculated based on four metrics as the geometric mean (equally weighted) of life expectancy, education, and gross income per capita, as follow^71^:

$$ HDI = \left( {I_{Health} *I_{Education} *I_{Income} } \right)^{1/3} $$

where the *Health* component—a long and healthy life—is based on estimates of life expectancy, the *Education* component—access to education—is calculated with both the expected years of schooling (for children at school entering age) and mean years of schooling (for adults aged 25 an older), and the *Income* component—a decent standard of living—is measured by Gross National Income per capita^66^. The values of each of the four metrics are first normalized to an index value of 0 to 1, and then, once each of the individual indices has been calculated, they are aggregated to calculate the HDI according to the previous formula. The dimension index is therefore 1 (i.e*.* achieves the maximum value) and it is 0 when is at the minimum value^71^. The HDI values at neighborhood level for the city of Porto Alegre were obtained from the Atlas of Human Development from the ObservaPOA (observapoa.com.br)^72^.

### Earth observation data

To investigate the environmental factors influencing mental health diagnosticated cases, a remote sensing approach based exclusively on high-resolution optical Earth observation data was used. We used the European Space Agency (ESA) global Land Use/Land Cover (LULC) data set WorldCover^45^ at 10 m resolution. This product is based on both Sentinel-1 and Sentinel-2 data, containing 11 classes: Tree cover, Shrubland, Grassland, Cropland, Built-up, Bare/sparse vegetation, Snow and Ice, Permanent water bodies, Herbaceous Wetland, Mangrove and Moss and lichen. Those classes are defined using the Land Cover Classification System (LCCS) developed by the United Nations (UN) Food and Agriculture Organization (FAO). We use the Tabulate Intersection tool from the Spatial Analysis toolbox in ArcGIS^69^ to calculate the percentage of each Land Use/Land Cover (LULC) variable within each neighborhood of our study area. These percentages values were used as independent variables in our models (Supplementary Table S1).

### Data analysis

We used a geospatial analysis to explore the spatial distribution and to identify the hotspot areas of mental health cases. ArcGIS version 10.8^69^ was used to perform the Spatial Global Autocorrelation (Global Moran’s I) to assess whether the Mental Health Index (MHI) exhibited a random distribution or was spatially clustered (i.e. indicating spatial autocorrelation)^73^. Then, we performed a HotSpot analysis (Getis-Ord Gi* Statistical) to identify significant hot spot and cold spot areas. Z-score and *p*-value were evaluated to assess the statistical significance of these clusters. A high GI* value indicates hot spot areas, while a low GI* value indicates cold spot areas^74^.

To detect the most important variable affecting the Mental Health Index (MHI), we used a Random Forest (RF) algorithm. This machine learning approach involves using multiple conditional classification trees to assess the significance of individual variables in predicting the outcome variable^75^. By employing a classification tree framework, this method enables the analysis of data without the need for assumptions typically necessary in traditional parametric tests, such as balanced designs and sample independence^76^ and was introduced by Breiman^77^. We used 80% of the data as the training data set and 20% as the test data set. The model goodness-of-fit and accuracy of the RF were measured using the Mean Squared Error (MSE), Root Mean Squared Error (RMSE) and Mean Absolute Error (MAE). Lower values in those metrics indicate a better model fit. The variable importance scores were estimated based on the statistics of the mean decrease in node impurity (IncNodePurity) when a variable is chosen to split a node, the higher the value of the mean increase in node purity, the greater the importance of the variable in the model^75^. To test the effects of predictors on our dependent variable, we used Polynomial Regression. To be able to model non-linear relationships, we fitted higher-order polynomial functions^78^. The fitted Polynomial Regression included predictors obtained from RF based on the most important variable and they were evaluated based on Akaike’s information criterion (AIC) and Bayesian Information Criterion (BIC) together with the Residual standard error^79^.

## Supplementary Information

Supplementary Table S1.

## Data Availability

Land Use/Land Cover data supporting this research is available via the ESA WorldCover managed by the European Space Agency at worldcover2020.esa.int. The Human Development Index data supporting this research is available via the Ob- servaPOA managed by the City Hall of Porto Alegre at observapoa.com.br. Mental Health data supporting this research are available from the City Hall of Porto Alegre and aren’t accessible to the public or research community without explicit approval. Please, contact the City Hall of Porto Alegre through the Health Surveillance Directorate of the city of Porto Alegre at evdant@portoalegre.rs.gov.br to discuss data and access requirements.
