# Supplementary material for: Earth observation data uncover green spaces’ role in mental health
Source: Sci Rep. 2024 Sep 9;14:20933. doi: 10.1038/s41598-024-72008-8 (PMC11384788; doi:10.1038/s41598-024-72008-8)
Supplement: Supplementary file 1 — Supplementary Table S1. [file 41598_2024_72008_MOESM1_ESM.docx]

**Supplementary Material**

This document contains the data from statistical analyses of the Land Use/Land Cover and The Human Development Index. It includes:

1 - Tables with descriptive statistics for Land Use/Land Cover percentages and The Human Development Index by neighborhoods.

S1. Percentages of each Land Use/Land Cover and The Human Development Index.

| **Tree cover (%)** | **Shrubland (%)** | **Grassland (%)** | **Cropland (%)** | **Built-up (%)** | **Sparse vegetation (%)** | **Water bodies (%)** | **Herbaceous wetland (%)** | **IDH** |
| --- | --- | --- | --- | --- | --- | --- | --- | --- |
| 55.04 | 0.199 | 11.203 | 0.100 | 33.374 | 0.045 | 0.000 | 0.033 | 0.843 |
| 82.60 | 0.032 | 8.684 | 0.032 | 8.402 | 0.186 | 0.000 | 0.003 | 0.683 |
| 16.56 | 0.000 | 24.257 | 2.795 | 46.033 | 1.170 | 0.139 | 9.023 | 0.765 |
| 55.86 | 0.000 | 6.834 | 0.059 | 3.399 | 0.026 | 2.348 | 31.127 | 0.659 |
| 14.77 | 0.000 | 0.325 | 0.000 | 84.881 | 0.026 | 0.000 | 0.000 | 0.935 |
| 11.46 | 0.000 | 2.105 | 0.000 | 85.647 | 0.000 | 0.620 | 0.123 | 0.935 |
| 28.56 | 0.000 | 0.620 | 0.000 | 70.063 | 0.739 | 0.000 | 0.000 | 0.935 |
| 46.89 | 0.000 | 34.704 | 0.015 | 12.923 | 0.070 | 1.949 | 3.346 | 0.714 |
| 78.23 | 0.010 | 6.328 | 0.095 | 15.228 | 0.105 | 0.000 | 0.000 | 0.733 |
| 31.63 | 0.000 | 15.329 | 0.212 | 52.689 | 0.132 | 0.000 | 0.004 | 0.890 |
| 42.15 | 0.002 | 32.611 | 16.316 | 2.163 | 0.059 | 0.902 | 5.734 | 0.714 |
| 5.56 | 0.000 | 0.967 | 0.000 | 93.470 | 0.000 | 0.000 | 0.000 | 0.935 |
| 14.71 | 0.000 | 0.088 | 0.000 | 85.164 | 0.021 | 0.000 | 0.000 | 0.777 |
| 18.79 | 0.004 | 0.535 | 0.074 | 80.597 | 0.000 | 0.000 | 0.000 | 0.797 |
| 57.73 | 0.228 | 14.978 | 1.352 | 25.620 | 0.033 | 0.000 | 0.058 | 0.797 |
| 73.12 | 0.025 | 12.126 | 0.026 | 13.350 | 1.308 | 0.048 | 0.000 | 0.733 |
| 27.59 | 0.000 | 2.623 | 0.136 | 69.494 | 0.042 | 0.103 | 0.000 | 0.797 |
| 6.61 | 0.000 | 2.170 | 0.009 | 90.158 | 0.303 | 0.504 | 0.061 | 0.935 |
| 54.25 | 0.006 | 35.080 | 0.756 | 8.491 | 0.099 | 0.349 | 0.969 | 0.714 |
| 16.80 | 0.000 | 0.825 | 0.021 | 82.316 | 0.041 | 0.000 | 0.000 | 0.777 |
| 6.82 | 0.000 | 0.025 | 0.000 | 93.127 | 0.029 | 0.000 | 0.000 | 0.935 |
| 36.84 | 0.016 | 12.728 | 0.026 | 50.279 | 0.101 | 0.000 | 0.000 | 0.777 |
| 18.26 | 0.000 | 6.345 | 0.764 | 73.905 | 0.690 | 0.000 | 0.000 | 0.779 |
| 18.02 | 0.002 | 8.497 | 2.172 | 67.778 | 2.518 | 0.413 | 0.497 | 0.809 |
| 9.61 | 0.000 | 0.161 | 0.010 | 89.899 | 0.301 | 0.000 | 0.000 | 0.890 |
| 40.25 | 0.000 | 5.517 | 0.572 | 53.131 | 0.055 | 0.340 | 0.074 | 0.843 |
| 72.04 | 0.000 | 23.431 | 1.730 | 1.758 | 0.075 | 0.236 | 0.686 | 0.714 |
| 5.00 | 0.000 | 8.140 | 0.095 | 83.230 | 1.017 | 1.134 | 1.195 | 0.765 |
| 51.56 | 0.000 | 4.282 | 0.025 | 41.957 | 0.646 | 1.415 | 0.112 | 0.935 |
| 3.81 | 0.028 | 2.916 | 0.056 | 92.197 | 0.408 | 0.444 | 0.024 | 0.935 |
| 44.28 | 0.000 | 8.473 | 0.000 | 47.196 | 0.044 | 0.000 | 0.000 | 0.733 |
| 40.22 | 0.111 | 7.399 | 0.039 | 50.547 | 0.126 | 1.324 | 0.137 | 0.843 |
| 25.13 | 0.000 | 0.209 | 0.000 | 74.657 | 0.000 | 0.000 | 0.000 | 0.890 |
| 23.66 | 0.008 | 10.413 | 0.263 | 62.545 | 0.475 | 0.147 | 2.438 | 0.765 |
| 49.05 | 0.022 | 16.899 | 0.732 | 32.394 | 0.447 | 0.004 | 0.451 | 0.843 |
| 12.09 | 0.000 | 0.000 | 0.000 | 87.908 | 0.000 | 0.000 | 0.000 | 0.935 |
| 34.52 | 0.000 | 2.901 | 0.047 | 61.872 | 0.256 | 0.290 | 0.032 | 0.843 |
| 32.34 | 0.000 | 4.238 | 0.000 | 62.850 | 0.038 | 0.424 | 0.087 | 0.935 |
| 39.52 | 0.013 | 5.186 | 0.064 | 54.929 | 0.272 | 0.008 | 0.000 | 0.777 |
| 27.47 | 0.000 | 2.727 | 0.263 | 69.422 | 0.044 | 0.009 | 0.000 | 0.777 |
| 28.18 | 0.000 | 4.201 | 0.662 | 66.599 | 0.077 | 0.278 | 0.000 | 0.890 |
| 11.32 | 0.000 | 5.240 | 0.000 | 80.792 | 0.102 | 0.928 | 1.621 | 0.890 |
| 40.69 | 0.000 | 0.619 | 0.000 | 58.671 | 0.000 | 0.000 | 0.000 | 0.843 |
| 18.41 | 0.000 | 5.875 | 0.084 | 75.217 | 0.390 | 0.000 | 0.000 | 0.779 |
| 11.29 | 0.000 | 5.631 | 0.000 | 82.922 | 0.120 | 0.000 | 0.000 | 0.638 |
| 16.26 | 0.000 | 2.082 | 0.067 | 81.192 | 0.388 | 0.000 | 0.008 | 0.890 |
| 46.44 | 0.000 | 1.785 | 0.039 | 51.585 | 0.064 | 0.071 | 0.000 | 0.777 |
| 13.06 | 0.000 | 15.001 | 0.973 | 69.236 | 1.459 | 0.000 | 0.261 | 0.890 |
| 76.14 | 0.002 | 19.143 | 0.754 | 3.731 | 0.035 | 0.034 | 0.148 | 0.714 |
| 47.91 | 0.002 | 34.821 | 1.657 | 5.316 | 0.038 | 1.376 | 8.778 | 0.714 |
| 65.86 | 0.001 | 13.674 | 0.314 | 19.603 | 0.132 | 0.251 | 0.112 | 0.683 |
| 12.39 | 0.000 | 1.788 | 0.233 | 85.480 | 0.074 | 0.000 | 0.000 | 0.747 |
| 9.29 | 0.000 | 1.283 | 0.013 | 88.585 | 0.039 | 0.625 | 0.142 | 0.935 |
| 23.58 | 0.000 | 0.572 | 0.000 | 75.534 | 0.000 | 0.238 | 0.057 | 0.935 |
| 20.66 | 0.000 | 0.000 | 0.000 | 79.343 | 0.000 | 0.000 | 0.000 | 0.935 |
| 53.79 | 0.036 | 15.199 | 0.004 | 30.664 | 0.271 | 0.000 | 0.000 | 0.777 |
| 35.16 | 0.000 | 12.249 | 0.482 | 51.609 | 0.355 | 0.042 | 0.004 | 0.638 |
| 1.83 | 0.000 | 1.111 | 0.144 | 95.536 | 0.595 | 0.661 | 0.033 | 0.765 |
| 45.69 | 0.298 | 10.611 | 0.040 | 43.342 | 0.011 | 0.000 | 0.000 | 0.797 |
| 27.35 | 0.000 | 10.159 | 0.618 | 61.313 | 0.536 | 0.000 | 0.000 | 0.779 |
| 19.61 | 0.007 | 3.218 | 0.063 | 76.837 | 0.065 | 0.171 | 0.022 | 0.764 |
| 12.20 | 0.000 | 0.499 | 0.000 | 87.302 | 0.000 | 0.000 | 0.000 | 0.890 |
| 26.25 | 0.000 | 10.984 | 0.952 | 61.721 | 0.061 | 0.000 | 0.026 | 0.779 |
| 71.95 | 0.000 | 1.667 | 0.098 | 23.204 | 0.199 | 1.868 | 0.521 | 0.843 |
| 25.23 | 0.000 | 0.415 | 0.011 | 74.110 | 0.055 | 0.140 | 0.021 | 0.935 |
| 81.13 | 0.003 | 12.604 | 0.105 | 6.144 | 0.011 | 0.000 | 0.000 | 0.685 |
| 63.23 | 0.001 | 19.406 | 0.707 | 13.197 | 0.048 | 0.898 | 2.409 | 0.714 |
| 21.09 | 0.014 | 21.544 | 1.961 | 49.171 | 1.578 | 2.548 | 1.725 | 0.935 |
| 55.18 | 0.012 | 14.349 | 0.229 | 29.594 | 0.538 | 0.043 | 0.048 | 0.685 |
| 17.90 | 0.000 | 0.133 | 0.116 | 81.796 | 0.044 | 0.000 | 0.000 | 0.935 |
| 16.94 | 0.000 | 6.500 | 0.232 | 75.630 | 0.497 | 0.000 | 0.079 | 0.779 |
| 13.69 | 0.000 | 3.267 | 0.000 | 81.835 | 0.000 | 1.009 | 0.198 | 0.935 |
| 4.96 | 0.000 | 1.412 | 0.000 | 93.406 | 0.172 | 0.000 | 0.011 | 0.890 |
| 15.54 | 0.001 | 12.939 | 0.580 | 62.959 | 0.324 | 0.000 | 7.619 | 0.729 |
| 34.00 | 0.000 | 3.071 | 0.112 | 62.747 | 0.026 | 0.000 | 0.000 | 0.747 |
| 6.17 | 0.000 | 1.171 | 0.000 | 91.799 | 0.005 | 0.576 | 0.280 | 0.935 |
| 24.24 | 0.000 | 0.597 | 0.000 | 75.120 | 0.000 | 0.000 | 0.000 | 0.764 |
| 9.90 | 0.001 | 14.039 | 8.609 | 36.763 | 0.507 | 0.911 | 29.215 | 0.729 |
| 52.03 | 0.000 | 20.435 | 0.029 | 17.294 | 0.023 | 4.672 | 5.384 | 0.843 |
| 79.36 | 0.000 | 18.511 | 0.828 | 0.822 | 0.025 | 0.097 | 0.307 | 0.714 |
| 2.89 | 0.000 | 0.446 | 0.000 | 96.041 | 0.194 | 0.346 | 0.000 | 0.765 |
| 7.25 | 0.000 | 0.868 | 0.000 | 91.826 | 0.028 | 0.000 | 0.000 | 0.890 |
| 10.98 | 0.000 | 0.548 | 0.062 | 88.344 | 0.041 | 0.000 | 0.021 | 0.890 |
| 87.62 | 0.000 | 3.825 | 0.001 | 8.538 | 0.000 | 0.000 | 0.000 | 0.843 |
| 56.11 | 0.017 | 7.099 | 0.186 | 36.564 | 0.010 | 0.000 | 0.000 | 0.797 |
| 27.35 | 0.000 | 1.102 | 0.000 | 70.506 | 0.013 | 0.779 | 0.101 | 0.843 |
| 27.09 | 0.000 | 2.428 | 0.000 | 70.180 | 0.300 | 0.000 | 0.000 | 0.777 |
| 37.62 | 0.000 | 1.677 | 0.027 | 58.844 | 0.044 | 1.230 | 0.141 | 0.843 |
| 67.72 | 0.000 | 0.076 | 0.000 | 30.314 | 0.611 | 1.138 | 0.000 | 0.843 |
| 15.74 | 0.000 | 0.680 | 0.160 | 83.360 | 0.033 | 0.000 | 0.000 | 0.890 |
| 14.08 | 0.000 | 0.416 | 0.000 | 85.479 | 0.010 | 0.000 | 0.000 | 0.777 |
| 10.54 | 0.000 | 0.205 | 0.000 | 89.179 | 0.033 | 0.000 | 0.000 | 0.764 |
| 58.25 | 0.000 | 12.719 | 0.447 | 28.423 | 0.155 | 0.001 | 0.007 | 0.764 |
| 30.66 | 0.194 | 5.580 | 0.012 | 60.225 | 2.720 | 0.601 | 0.000 | 0.764 |
